# Ulcerative colitis complicated by primary sclerosing cholangitis and autoimmune hepatitis overlap syndrome: a case report and literature review

**DOI:** 10.3389/fimmu.2023.1132072

**Published:** 2023-05-09

**Authors:** Xinhe Zhang, Xuyong Lin, Xuedan Li, Lin Guan, Yiling Li, Ningning Wang

**Affiliations:** ^1^ Gastroenterology Department, The First Hospital of China Medical University, Shenyang, Liaoning, China; ^2^ Department of Pathology, The First Hospital of China Medical University, Shenyang, Liaoning, China; ^3^ Radiology Department, The First Hospital of China Medical University, Shenyang, Liaoning, China

**Keywords:** primary sclerosing cholangitis, autoimmune hepatitis, ulcerative colitis, overlap syndrome, UDCA

## Abstract

Primary sclerosing cholangitis (PSC), autoimmune hepatitis (AIH), and ulcerative colitis (UC) are immune diseases of the digestive system. Some patients develop overlap syndrome, the presentation of two or more of the clinical, biochemical, immunological, and histological features of these conditions simultaneously or sequentially. The incidence of UC in PSC-AIH overlap syndrome is as high as 50%. In contrast, PSC-AIH overlap syndrome is rare in UC patients. However, because it has a low prevalence and has been studied in less detail, PSC is often misdiagnosed as primary biliary cholangitis (PBC) in its early stage. Herein, we reported a case of a 38-year-old male patient who presented to a clinician in 2014 with irregular bowel habits. A colonoscopy suggested UC. In 2016, the patient was found to have abnormal liver function and was diagnosed with PBC by pathology. He was treated with ursodeoxycholic acid (UDCA) but this had no effect on his liver function. Additional liver biopsies in 2018 indicated PBC-AIH overlap syndrome. The patient refused hormone therapy for personal reasons. Following UDCA monotherapy, his liver function remained abnormal. The patient was reexamined after repeated abnormal liver function tests and bowel symptoms. Systematic laboratory testing, imaging diagnosis, colonoscopy, liver biopsy, and various pathological examinations conducted in 2021 were used to diagnose the patient with PSC-AIH-UC overlap syndrome. He was treated with various drugs, including UDCA, methylprednisolone, mycophenolate mofetil, and mesalazine. His liver function improved significantly after treatment and follow-up is ongoing. Our case report highlights the need to raise awareness about rare and difficult-to-diagnose clinical disorders.

## Introduction

Immune dysfunction can lead to a number of different autoimmune diseases. There are two main types of gastrointestinal involvement, including inflammatory bowel disease (IBD) and autoimmune liver disease (ALD) (1). IBD includes ulcerative colitis (UC) and Crohn’s disease (CD), while ALD includes autoimmune hepatitis (AIH), primary biliary cholangitis (PBC), and primary sclerosing cholangitis (PSC) (2). These conditions can happen both independently and together. Two or more diseases appear simultaneously or sequentially to form overlap syndrome, such as PBC-AIH, PSC-AIH, and PSC-UC. PSC-AIH overlap syndrome primarily affects children, adolescents, and young men. Imaging features of the bile duct resemble PSC while the clinical, biochemical, and histological features are consistent with AIH (3). The incidence of IBD is as high as 40–50% in PSC-AIH overlap syndrome patients (4); however, PSC-AIH overlap syndrome is rare in IBD patients (5). Herein, we described a 38-year-old male UC patient, who received a diagnosis of PBC after 2 years because of abnormal liver function. However, his liver function remained abnormal after UDCA treatment. After 5 years, a diagnosis of PSC-AIH-UC overlap syndrome was confirmed by a systematic and detailed examination. The diagnostic pathway informs the development of more accurate clinical diagnostics.

## Case report

In 2014, a 38-year-old male patient underwent a colonoscopy because of irregular bowel habits and increased stool frequency lasting for 1 month. The colonoscopy showed that the distal ileum was found to be smooth, and the colonic mucosa was congested with hematomas. Punctate ulcers, erosions, and multiple bleeding spots from the anus to the ileocecal area were observed, which is indicative of UC under the colonoscope. However, the patient voluntarily discontinued mesalazine treatment after 1 month.

In 2016, the patient was found to have significantly elevated liver function indicators during the physical examination (**Table 1**). He underwent a liver biopsy at the local hospital and was given a pathological diagnosis of “PBC” through description by himself. So he was orally treated with 15 mg/kg/d ursodeoxycholic acid (UDCA). Intermittent follow-up revealed that his liver function results were still abnormal.

**Table 1:** Changes in liver function and immunoglobulin levels over time.

	ALT (9–50U/L)	AST (15–40U/L)	ALP (45–125U/L)	GGT (10–60U/L)	IgG (7–17U/L)	IgG4 (0.03–2.01g/L)	IgM (0.40–2.30U/L)
2014.12.05	43	52	101	53	16.4	NA	1.2
2016.12.14	38	20	577	268	17.4	NA	1.1
2018.12.25	57	128	316	237	14.5	1.77	0.83
2021.01.23	236	132	415	199	NA	NA	NA
2021.06.05	89	64	332	186	21.36	NA	1.56
2021.08.25 (hospital stay)	63	64	342	260	29.57	2.68	1.33

NA, not applicable.

In 2018, the patient experienced fatigue and poor appetite. A medical visit revealed that he still had abnormal liver function results (**Table 1**). An antinuclear antibody (ANA) test was positive. Imaging indicated chronic hepatic damage and slight dilatation of the intrahepatic bile ducts. Then a secondary liver biopsy was performed at the local hospital and revealed that a portion of the portal area was enlarged and there was evidence of lymphocyte, plasma cell, and eosinophil infiltration. Mild interface inflammation, focal bile duct epithelial deformation, focal peribiliary fibrosis, and focal necrosis of hepatocytes were also observed. The patient was diagnosed with focal atypical PBC-AIH overlap syndrome using these findings. UDCA monotherapy was continued after the patient declined corticosteroid treatment for personal reasons. His liver function continued to be abnormal at subsequent follow-up appointments.

In 2021, the patient was admitted to our hospital due to recurrent abnormal liver function. After detailed questioning of his medical history, he reported fatigue, increased frequency of bowel habits (3–4 times/day), and unformed stool (without mucus, pus, or blood). He had no fever, jaundice, itchy skin, joint pain, rash, abdominal pain, or other symptoms. He denied alcohol use, viral hepatitis infection, or family history. No obvious abnormalities were observed during the physical examination, including no yellowing of the skin and sclera, and no liver palms or spider nevus.

**Figure 1:**
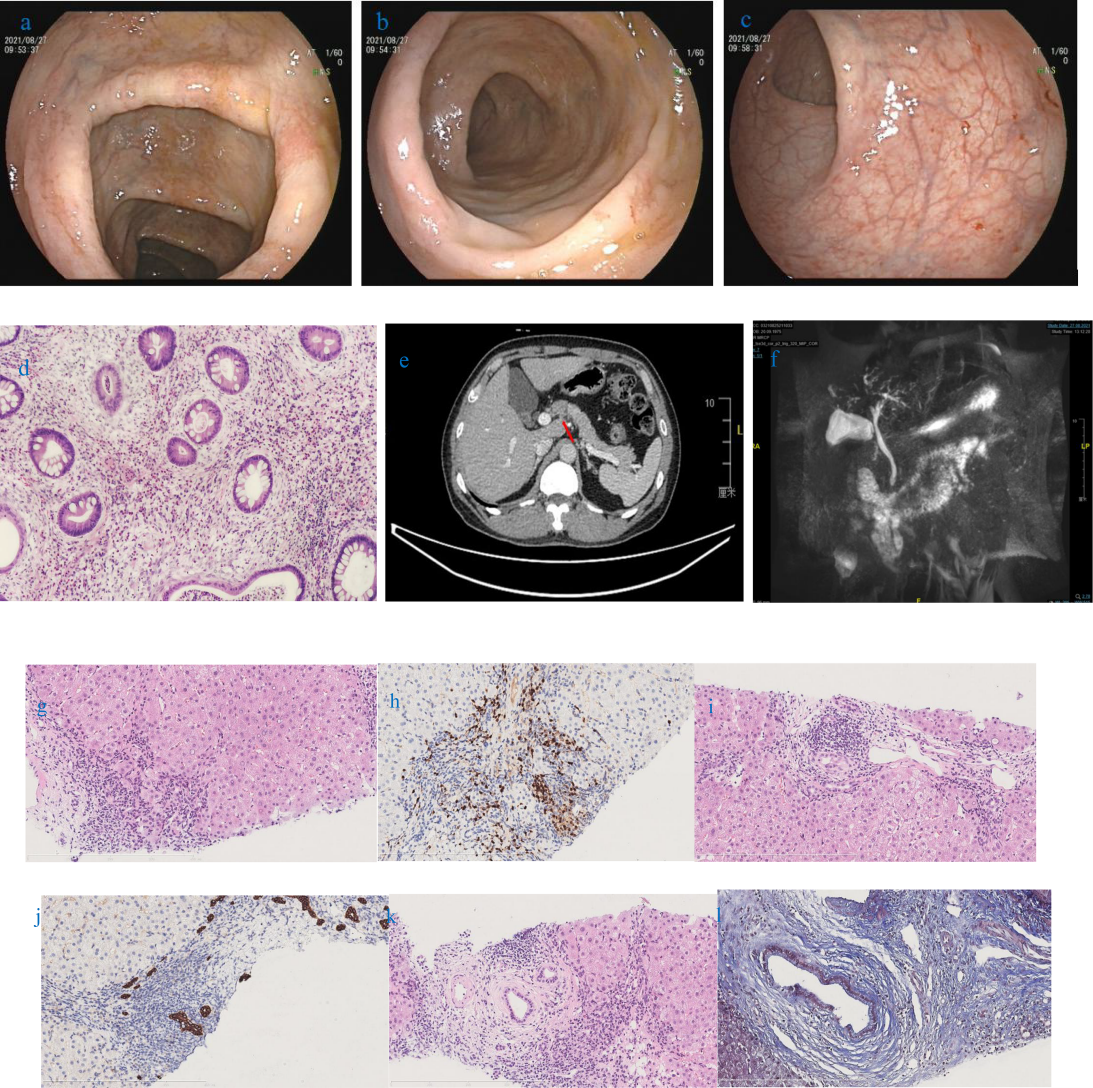
**(A)** Ascending colon; **(B)** transverse colon; **(C)** Rectum, **(D)** Pathology. Colonoscopy images show the ascending colon mucosa scattered in sheet congestion edema with a granular surface. The transverse colon mucosa are scattered in sheet erythema. The descending colon and sigmoid mucosa are smooth. Erosive, ulceration, loss of vascular markings, or mucosal friability are not observed in the patient. **(E)** An enhanced CT scan of the liver shows that the liver surface is not smooth and the intrahepatic bile ducts are slightly dilated. **(F)** MRCP shows segmental dilatation of the intrahepatic bile ducts. **(G)** Moderate interface inflammation and significant lymphoplasmacytic infiltration; **(H)** Immunohistochemistry MUM1 (+); **(I)** Some bile ducts are surrounded by lymphocytes and phagocytes, and lymphocytes involved in the bile duct; **(J)** Immunohistochemistry CK7 (+); **(K)** Concentric fibrotic changes around the large bile duct with scattered plasma cell infiltration, and bile duct hyperplasia was seen in the converduct area; **(L)** MASSON staining showed concentric fibrosis changes and deformation of the bile duct.

In terms of abnormal liver function, we reexamined the relevant test. Liver function indicators were significantly increased (**Table 1**) and albumin had a mildly reduced level of 31.6 g/L. No hepatitis markers were abnormal. We excluded drugs and hepatitis as the causes while considering the influence of immune factors. Thus, we considered whether it was related to ALD, IgG4-related disease, or other diseases. We then carried out a serological examination of immunoglobulin and related antibodies. ANA (Nuclear homogeneous type 1:320+, Cytoplasmic granular type 1:160+), pANCA, and PR3-ANCA antibodies (chemiluminescence method: 264.3CU) were positive, while AMA-M2, M2-3E, sp100, gp210, ASMA, LKM, LC-1, SLA/LP, Ro-52, Scl-70 antibodies were negative. IgG, IgG4, and IgM levels were all higher than the upper limit of normal, and serum gamma protein electrophoresis was 36.3%. Related imaging examinations were also completed. An ultrasound of the liver showed cirrhosis and dilation of the portal venous system. An enhanced CT scan of the liver and the MRCP findings are shown in **Figure 1**. We performed a third liver biopsy. A total of 14 small and medium portal areas were observed in the liver biopsy tissue and the lobular structure was disordered. The primary lesions showed a marked expansion of the portal area, moderate to severe inflammatory cell infiltration consisting primarily of mononuclear cells, moderate interface inflammation, and coexistence of small bile duct hyperplasia and deletion. Onion skin-like fibrosis was seen around some small bile ducts, different degrees of thin bile duct reaction were observed around the portal area, and marginal bile ducts were present. Individual plasma cells were positive for IgG4 (the ratio of IgG4/IgG is about 5%). A centrilobular-portal bridging necrosis zone and focal necrosis were seen in the lobule and mild inflammatory cell infiltration was present in the sinus. The pathological morphology was suggestive of PSC-AIH overlap syndrome (G3-4,S3) (**Figure 1**).

In terms of abnormal stool, a fecal culture did not reveal evidence of bacteria or fungal infection. A colonoscopy revealed changes in colonic inflammation (predominantly in the right colon) (**Figure 1**). Pathology in the colon showed chronic inflammation (plasmacytosis and lymphocyte) of the mucosa, erosion, gland atrophy, crypt distortion, and cryptitis (**Figure 1**).

Due to the elevation of IgG4 levels, the patient also underwent an ultrasound of the superficial glands and lymph nodes, which revealed coarse echoes of the bilateral submandibular, parotid, and lacrimal glands, and grade 2 echoes of the bilateral cervical, double supraclavicular, double axillary, and double inguinal lymph nodes. Pathology of the submandibular gland showed semi-scattered plasma cells without IgG4 lymphocyte infiltration. Thus, we excluded the diagnosis of IgG4-related disease.

The patient was finally diagnosed with PSC-AIH-UC overlap syndrome and prescribed oral 0.5 g UDCA twice a day, 40 mg methylprednisolone once a day, 0.25 g mycophenolate mofetil three times a day, and 1.0 g mesalazine three times a day. These drugs were used to adjust the patient’s gut flora, protect his stomach, and supply the calcium needed to prevent hormone-related side effects. After the patient was discharged from the hospital later that month, he underwent regular follow-ups. His symptoms and liver function test results improved significantly. At his last follow-up on March 19, 2022, the patient’s AST, ALT, IgG4, and IgG levels had all returned to normal ranges, and ALP and GGT levels had decreased significantly. The drug dosage was adjusted according to the patient’s indicators. He now receives oral 1.25 g UDCA once a day, 12 mg methylprednisolone once a day, and 1.0 g mycophenolate mofetil once a day and is still receiving follow-up.

## Discussion

This study describes a male patient with a final diagnosis of PSC-AIH-UC overlap syndrome over seven years. It is worth discussing that the first two liver biopsy results suggested PBC because it is unusual for a patient to have both PBC and UC, especially as the patient is male and AMA, SP100, and GP210 tests were all negative. Since the first reported case of PBC and UC in 1985, only 20–30 sporadic cases have been reported (6). As a result, there is a lower incidence of PBC-AIH overlap syndrome than PBC alone and there are no reports of PBC-AIH-UC overlap syndrome. Thus, an additional liver biopsy was performed on the patient. ALP and GGT were both elevated, high or positive PR3-ANCA was seen, and MRCP revealed segmental dilatation of the intrahepatic bile ducts. Pathology showed onion skin-like fibrosis around the bile ducts, bile duct absence, and hyperplasia, indicating that the patient met the characteristics of PSC. He also had increased transaminase and IgG levels, moderate interface inflammation, bridging necrosis in most portal areas, and abnormal infiltration of plasma cells, which met the characteristics of AIH. The levels of inflammation and fibrosis were higher in this biopsy than in the one conducted in 2018. After the final diagnosis was made, the patient’s prior pathology films were re-evaluated. We think he should have been previously diagnosed with PSC and not PBC, which may have occurred due to an insufficient understanding of PSC at the time.

PSC is the most common complication of cholestasis and is closely related to UC. The incidence of IBD in PSC patients can be as high as 70%–86%, of whom more than 75% are complicated by UC (7). In contrast, PSC is rare in IBD patients (8). The latest meta-analysis (9) reported that the combined prevalence of PSC in IBD patients was 2.16% and was the highest in South America and the lowest in Southeast Asia. The combined prevalence of patients with UC, CD, and unclassified IBD were 2.47%, 0.96%, and 5.01%, respectively (10). PSC-IBD overlap syndrome is an independent disease entity. When the two diseases coexist, UC exhibits a characteristic disease phenotype. Compared with UC patients, PSC-UC patients have pancolitis, heavier right-sided colonic inflammation, rectal parenchymal and retrograde ileitis, and a higher incidence of colorectal neoplasia (11). These patients also have increased right colonic histological activity and decreased rectal histological activity. Prolonged inflammatory disease activity or the accumulation of secondary bile acids may explain the predominance of right-sided tumor formation among PSC-UC patients (12). There are no effective drug therapies for PSC, so patients are primarily treated with UDCA. UDCA (17-23mg.kg^-1^.d^-1^) can improve liver function, including liver fibrosis and biliary imaging. However, there is no strong evidence for improved survival and prognosis (7). Early clinical trials have found that cyclosporine can alleviate colonic lesions in PSC-UC patients (13). There are also reports that oral vancomycin can induce gut microbial transformation in this patient population (14), but these drugs will need to be further studied in long-term trials.

A recent study found that among 3,684 UC patients, the prevalence of AIH was 0.24% (5). Patients with IBD had a higher prevalence of AIH, with odds ratios of 7.8 and 17.9 for men and women, respectively (5). These patients are more likely to fail treatment and develop cirrhosis (15). A proposed mechanism for the relationship between UC and AIH is the disruption of colonic permeability and the activation of an immune response in the liver (16). UC patients have a defective intestinal mucosal protective barrier that increases permeability and intestinal exposure to various toxins. The liver absorbs high levels of toxins from the intestine through the portal vein, activating the immune system and causing liver cell damage (17).

AIH is found in 1.4%–17% of PSC patients (18). Most patients with PSC-AIH overlap syndrome are treated with UDCA + steroids + azathioprine or an immunosuppressive agent, which can improve the condition in the short term (19). A meta-analysis found that 48 (44.44%) of 109 PSC-AIH overlap syndrome patients had IBD (4), of whom 68.08% were diagnosed with UC (20). Most of these patients were children. Hepatobiliary diseases are closely related to IBD and hepatobiliary dysfunction is also a common extraintestinal manifestation of UC (18). PSC, AIH, and UC are immune-related diseases with several genetic and environmental risk factors. While common susceptibility genes have not yet been identified, microbial involvement appears to link these diseases. The concept of a “gut-liver axis” has been widely accepted (21). Intestinal flora participate in the normal metabolism of bile acids by synthesizing bile acid hydrolase and steroid dehydrogenase (22). Abnormal bile acid metabolism can inhibit intestinal bacterial growth and reduce intestinal function by destroying the integrity of cell membranes, damaging DNA, and inducing protein denaturation and inactivation (23). Bacteria and potentially toxic products enter the liver *via* the gut-liver axis to stimulate the secretion of pro-inflammatory cytokines, resulting in immune disorders and triggering fibrosis by stimulating hepatic stellate cells (24).

**Table 2** describes cases of PSC-AIH-UC overlap syndrome reported in research articles (20, 25–30). The case reported by the current study highlights four points for consideration. First, multiple immune diseases can coexist, which may reflect a common pathogenic pathway. However, this also increases the complexity of disease diagnosis. Our case involves the differentiation of PBC and PSC. While both are cholestasis diseases, PBC mainly involves small bile ducts and PSC primarily involves bold ducts. AMAs are present in PBC patients, while ANA, pANCA, SMA, PR3-ANCA, and other autoantibodies are found in PSC patients (31). MRCP is very important for the diagnosis of PSC, which can reveal limited or diffuse bile duct stricture. However, cholangiography lacks specific features for the diagnosis of PBC (32). The most important indication of this disorder is pathological changes in the liver. PBC indicates non-suppurative cholangitis, while PSC has no specificity in its early stage, only biliary tract injury, and evidence of fibrosis at the late stage (33). Second, patients with overlapping immune diseases may have mild clinical manifestations. Clinical characteristics may be inconsistent with those found in the predisposed population. Therefore, it is necessary to focus on evidence of other immune diseases when diagnosing an immune disease. Third, the use of multiple clinical disciplines is very important for the diagnosis of these diseases. Liver histology plays an important role in liver-specific immune diseases. Fourth, the case described here was accompanied by an increase in IgG4, which is characteristic of IgG4-associated sclerosing cholangitis (IgG4-SC). However, 9%–27% of PSC patients also have elevated serum IgG4 antibodies (34). In addition, the imaging manifestations of PSC and IgG4-SC were similar, which made identification more challenging. We combined imaging and pathology of the liver and the submandibular gland and a colonoscopy to exclude the diagnosis of IgG4-SC in this patient. Thus, special attention should be paid to the identification of IgG4-SC and PSC patients with elevated IgG4.

**Table 2:** Summary of systematically reviewed clinical cases of PSC-AIH-UC overlap syndrome.

Author	Age	Gender	Clinical presentation	Diagnosis	Antibodies	Complication	Treatment	Outcome
Takanori Suzuki (25)	28	male	abnormal liver function, abdominal pain, bloody stool .	PSC (2016); UC (2019); PSC-AIH-UC (2021)	ANA ASMA	None	UDCA Azathioprine prednisone, ustekinumab	Recovery
Hon Yan Ng (26)	12	Female	abnormal liver function	PSC-AIH-UC (2021)	ASMA	multifocal osteomyelitis	Prednisone Azathioprine UDCA	Recovery
Vinícius Remus Ballotin (20)	22	Female	abdominal pain, jaundice, choluria, and acholia	PSC-AIH-UC (2020)	ANA	None	corticosteroids, azathioprine, ursodeoxycholic acid, and mesalamine	Recovery
Erling Peter Larsen (27)	10	male	weight loss, upper abdominal pain, vomiting, and diarrhea	PSC-AIH-UC (2012)	ASMA pANCA	None	Prednisolone UDCA azathioprine	Recovery
Aneta Nalepa (28)	15	male	abnormal liver function, diarrhea, abdominal pain	AIH (2008) PSC-AIH (2010) PSC-AIH-UC (2013)	ANA ASMA	None	corticosteroid and azathioprine UDCA	Deterioration and liver transplantation
J Koskinas (29)	18	male	fever	AIH PSC-AIH-UC(Two years later)	ANA	pyoderma gangrenosum	UDCA	liver transplantation
Miaoyu Jing (30)	55	male	Diarrhea	PSC-AIH-UC (2022)	AMA-M2	PBC	Prednisolone UDCA mesalamine	–

## Data availability statement

The original contributions presented in the study are included in the article/supplementary material. Further inquiries can be directed to the corresponding author.

## Ethics statement

Written informed consent was obtained from the individual for the publication of any potentially identifiable images or data included in this article. The studies involving human participants were reviewed and approved by the First Affiliated Hospital of China Medical University. The patients provided written informed consent to participate in this study.

## Author contributions

YL designed the study; XZ wrote the original draft; YL and NW collected the case; XYL, XDL provided the photo; YL, NW, LG reviewed and edited; All authors read, revised and approved the final manuscript.
